# Blood vessel remodeling in pig ovarian follicles during the periovulatory period: an immunohistochemistry and SEM-corrosion casting study

**DOI:** 10.1186/1477-7827-7-72

**Published:** 2009-07-16

**Authors:** Alessandra Martelli, Maria Grazia Palmerini, Valentina Russo, Carlo Rinaldi, Nicola Bernabò, Oriana Di Giacinto, Paolo Berardinelli, Stefania Annarita Nottola, Guido Macchiarelli, Barbara Barboni

**Affiliations:** 1 Department of Comparative Biomedical Sciences, University of Teramo, Piazza A. Moro, 45, 64100 Teramo, Italy; 2 Department of Health Sciences, Faculty of Medicine, University of L'Aquila, Italy; 3 Department of Anatomy, Sapienza University, Rome, Italy

## Abstract

**Background:**

The present research aims to describe the process of vascular readjustment occurring in pig ovary during the periovulatory phase (from LH surge to ovulation) that drives the transformation of the follicle, a limited blood supplied structure, into the corpus luteum, a highly vascularised endocrine gland required to maintain high levels of progesterone in pregnancy. The swine model was chosen because it is characterized by a long periovulatory window (about 40–44 hrs-similar to human) that permits to recover follicles at a precise endocrinological timing.

**Methods:**

By validated hormonal protocol (eCG+hCG), able to mimic the physiologic gonadotropin stimulation, preovulatory follicles (PreOFs, 60 h-eCG), follicles in the middle (early periovulatory follicles, EPerOFs, 18 h-hCG) or late (LPerOFs, 36 h-hCG) periovulatory phase were isolated from prepubertal gilts. To understand the angiogenic process, morphological/morphometrical analyses were performed by combining immunohistochemistry (IHC) and SEM of vascular corrosion casts (VCC) techniques.

**Results:**

PreOFs showed a vascular plexus with proliferating endothelial cells (EPI). This plexus was characterized by a dense inner capillary network, with angiogenic figures, connected to the outer network by anastomotic vessels (arterioles and venules of the middle network). EPerOFs decreased their EPI, blood vessel extension in the outer network, and evidenced a reduced compactness of blood vessels. In LPerOFs, a rapid neovascularization was associated to an intensive tissue remodeling: the follicle acquired an undulated aspect presenting arterioles/venules near the basal membrane, increased vascular extension by EPI, sprouting and non-sprouting angiogenesis.

The analysis of vascular geometric relations and branching angles evidenced similar values at all stages.

**Conclusion:**

These data allow us to hypothesize that EPerOFs are in a quiescent status. LPerOFs represent the "metamorphic" follicles that rapidly turn-on angiogenesis to sustain a successful corpus luteum formation. Particularly, it is interesting to underlie that the non-sprouting angiogenesis, typical of structures in rapid neovascularization, occurred only in the LPerOFs. Moreover, vascular geometric relations showed as blood vessel remodeling occurs with the "maximum output and the minimum energetic expense".

This knowledge will allow to better understand the mechanisms regulating the reproductive success and to clarify the complex physiological angiogenic process in adult tissues.

## Background

Folliculogenesis is a complex process involving dramatic functional and morphological differentiation of granulosa and theca cell layers. Although the process of follicle recruitment occurs cyclically, the final stage of development is physiologically reached only by a very small number of growing ovarian follicles [1]. These dramatic changes in tissue morphology and activity necessitate of significant changes in the microvascular extension.

Several functional and morphological studies were performed on ovarian angiogenesis, demonstrating the presence of active ovarian angiogenic factors, likely related to folliculogenesis and its gonadotropin stimulus [2-5]. In addition, numerous paracrine and autocrine factors are locally secreted under endocrine gonadotropin stimulus and may up- or down-regulate ovarian follicular angiogenesis [2,5,6]. Moreover, measurements of ovarian blood flow in mammals, using pulsed-Doppler technology, revealed an increased flow to the ovary containing the dominant follicle. In fact, in the dominant follicle before ovulation, an increased peak of flow velocity with an increasing follicular size and high vascularity, has been detected [7,8]. In particular, the perifollicular capillary network in the theca showed marked changes in and around the LH surge as increased blood vessels, vascular lumina, permeability of capillary walls and blood extravasion into the pericapillary stroma. These vascular changes cause edema of the theca first and of the entire follicle then, a condition that persists up to the follicular rupture [1]. These dynamic processes observed during the follicular-luteal transition, involve biochemical and morphological changes in the preovulatory follicles that, after the LH surge, will become periovulatory. Modifications also include the differentiation of theca and granulosa cells into luteal cells, tissue remodeling and growth, a switch in steroidogenesis, and an increase in the progesterone production. In order to meet these demands, the growth of blood vessels and the establishment of a blood supply (angiogenesis) is essential [2]. Despite numerous morphological studies have analyzed the distribution and the cyclic rearrangement of the ovarian blood vessels, in dissimilar experimental conditions and in different mammals [9-12], only few information is available on the physiological angiogenesis in the periovulatory structures, and in particular in follicles recovered after a precise endocrinological timing.

In our previous investigations we have analyzed the expression of vascular endothelial growth factor (VEGF), the main angiogenic factor, to evaluate its biological effects in pig ovarian follicles during the periovulatory period (from LH surge to ovulation) [6]. Instead, this research was designed to describe during the same phase the evolution of ovarian vascular remodeling that permits the transformation of the follicle, a limited blood supplied structure, into the corpus luteum, a highly vascularised endocrine gland. To this aim, in swine individual follicles, the standard histological approach was conducted in parallel to the scanning electron microscopy (SEM) of vascular corrosion cast (VCC) technique, which seems to be the best method to study angiogenesis, a phenomena evolving in a three-dimensional pattern and morphologically dynamic [9-14]. Moreover, by SEM of VCC, subtle ultrastructural details, and different structural conformations can be described [13,14]. In fact, VCC will allow to observe in pig periovulatory follicles sprouting angiogenesis, and eventually non-sprouting angiogenesis that does not require the immediate proliferation of endothelial cells but rather the rearrangement and plastic remodeling of existing ones. Furthermore, the great depth of focus of the SEM and the subsequent morphometry provide detailed quantitative data on blood vessels, such as diameters and branching degrees [14,15]. Indeed, it is important to emphasize that the vascular branching is related to optimality criteria like minimization of pumping energy or of building materials [16].

The choice of the pig model has been a consequence of its long periovulatory interval (40–44 h, similar to human), useful to study angiogenesis at a precise phase of follicle development by applying a validated hormonal treatment [5,6,17]. Spatial blood vessel distribution in the follicular wall has been visualized and the identification and quantification of angiogenesis has been possible by appreciating follicular vascular network, proliferating endothelial cells and sites of budding, sprouting, splitting (by transcapillary pillars or posts of extracellular matrix), and intussusception. The results obtained show that immediately after the LH-like surge, follicles are characterized by basal levels of angiogenesis that close up the follicular phase starting their metamorphosis for the next stage. In fact, the next structure (follicle close to ovulation) represents the metamorphosed follicle that turns-on its angiogenic activity, by sprouting and particularly by non-sprouting angiogenesis, to sustain a successful corpus luteum formation. The analysis of branching degrees allowed to postulate that this periovulatory follicular metamorphosis is driven by the balance between the principles of optimality and the biological needs and, thus, it occurs with a low energetic expense.

## Methods

### Experimental protocol and ovarian collection

Fifteen prepubertal Large White gilts, about six month old, with a weight of 93.7 ± 4.9 Kg (mean ± SD), were injected i.m. with a single dose of 1250 IU of eCG (Folligon, Intervet, Holland) to promote follicular growth in 60–72 h. Ovulation was induced with hCG (Corulon, Intervet) treatment (750 IU, i.m.) carried out 60 h later. The treated animals were divided in three groups of 5 animals each:

- Group 1: preovulatory follicles, 60 h after eCG (PreOFs);

- Group 2: early periovulatory follicles, 18 h after hCG (EPerOFs);

- Group 3: late periovulatory follicles, 36 h after hCG (LPerOFs).

Ovaries were recovered according to Martelli et al. [6]. All the protocols had prior approval of the Ethical Committee of the University of Teramo.

One ovary of each animal, immediately after removal, was processed for light microscopy [LM, [5,6]]. The contra lateral ovary was prepared for corrosion casts [10,12].

In this study only healthy follicles [5] with a diameter of > 6 mm were analyzed.

### Histological investigation and analysis

In the laboratory, each ovary was divided in 3–5 portions. After the dehydration step, each tissue portion was embedded in paraffin-wax. Serial paraffin sections of 5 μm thickness were collected on poly-L-lysine-coated slides and sequentially processed for investigations. In detail, each follicle was subjected, at least in double, to the following morphological and morphometrical analyses:

• hematoxylin-eosin (HE) staining to identify healthy follicles with diameter of > 6 mm;

• immunohistochemistry (IHC) for the endothelial marker von Willenbrand Factor (vWF) [6];

• double IHC (dIHC) technique for vWF and Ki-67 antigen, a cell proliferation marker [6];

• dIHC technique for vWF and α-SMA, a molecular marker of perivascular cells [18].

Normal goat serum for the vWF IHC, vWF and α-SMA dIHC or the TNB blocking buffer for the vWF and Ki-67 dIHC were used in place of the primary antisera as negative control. All controls performed were negative. As positive controls, human breast carcinoma tissue samples were used for vWF and Ki-67 antigen [5,6,19]. While, bovine aorta tissue collected at the slaughterhouse was used as the positive control for α-SMA [6]. The specificity of the double immunostaining was verified by localizing each antigen separately.

Morphological analyses were performed with an Axioscop 2plus epifluorescence microscope (Zeiss, Oberkochen, Germany) equipped with a cooled color charge coupled device camera (CCD; Axiovision Cam, Zeiss) interfaced to a computer workstation and provided with an interactive and automatic image analyzer (Axiovision, Zeiss).

Morphometrical analysis on tissue sections was performed using an image analysis system linked to a CCD (Zeiss) and the data were processed using a KS300 computed image analysis system (Zeiss).

#### HE and follicles identification

Tissue sections were stained with hematoxylin (Merk, Darmstadt, Germany) for 5 minutes, followed by a wash in water and acetic alcohol before staining with eosin (Merk) for 20 seconds. After dehydrating in ascending concentrations of ethanol, and then in xylene, sections were mounted.

On two or more HE sections, mean follicular diameter was calculated using the KS300 computed image analysis system (Zeiss), set to measure two diameters of the follicle section at right angles, and only symmetrical follicles (right angle cross sections within 10% of each other) were considered. Follicles were definitively judged as healthy when they showed a regular-shaped oocyte, surrounded by granulosa cells regularly apposed on an intact basement membrane, as well as granulosa cell nuclei without signs of pycnosis [2,5,6]. Follicles not fulfilling these criteria were classified as unsuitable for analyses.

#### vWF

This immunostaining was performed according to Martelli et al. [6]. The vascular area (VA) was given by the extension of vWF-positive area in μm^2^/15000 μm^2 ^and the results were expressed as mean values ± S.D. for the number of follicles analyzed within each follicular stage [5,6,19].

#### Ki-67 and vWF

The dIHC for Ki-67 and vWF was performed according to Martelli et al. [6]. For the morphometrical analysis, tissue sections were analyzed under 400× magnification. The quantification of the digitized fluorescent signals was completed by using a semi-automated algorithm by the image analysis system KS300. A guided program (macro for KS300) was created to count: 1) the number of proliferating endothelial cells (dual-stained cells for Ki-67-and vWF), 2) the number of proliferating theca cells (green-stained cells), 3) the total number (blue stained cells) of theca cells, inside a fixed area of 15000 μm^2 ^of the theca compartment. The endothelial proliferation index (EPI) was calculated as the percentage of proliferating endothelial cells (dual-stained cells for Ki-67-and vWF) on the total number of theca cells (blue stained cells) [19-21]. The whole area of the theca compartment was measured and the results expressed as mean values ± S.D. for the number of follicles analyzed within each follicular stage [19,21]. In order to eliminate the background due to the red blood cell autofluorescence from the quantitative analyses, the sections were re-stained with HE and micrographs of the same fluorescent fields were performed to identify and subtract the red blood cells within each blood vessel analyzed [6].

#### α-SMA and vWF

This dIHC was performed according to Martelli et al. [6] and Grazul-Bilska et al. [18]. Briefly, tissue sections, after mouse anti-α-SMA antibody [6], were incubated with biotinylated anti-goat secondary antibody (1:100 in PBS; Chemicon) 1 h at room temperature (RT) and avidin-biotin-peroxidase complex (Vectastain ABC kit) for 1 hr at RT. The immunocomplexes were then detected using 3, 3'diaminobenzidine (DAB; DBH Laboratory Supplies) by ammonium nickel sulphate method [5]. After washing in PBS, tissue sections were re-incubated with normal goat serum diluted 20% in PBS for 60 min and then with a rabbit anti-vWF antibody (diluted 1:400 PBS/BSA1%, Dako) RT-over night. After PBS washing, a secondary antibody anti-rabbit biotinylated (1:100 in PBS; Chemicon) was used for 1 hr at RT. After the ABC kit and triple PBS washing, the end-products of reaction were labeled by 0.05% DAB diluted in PBS plus 0.03% hydrogen peroxide, marking in brown the endothelial cells.

### Casting and scanning electron microscopy

The ovarian artery was perfused with a heparinized saline solution (RT), and then with Mercox^® ^resin (Okenshoji, Tokyo) [10,12]. The samples were air dried, mounted on aluminum stubs, and coated with platinum. Ovaries were analyzed by LM to identify the vascular plexuses with a diameter > 6000 μm. Once the structures of interest were localized the observations continued to the SEM performed at a low accelerating voltage (3–12 kV) in Hitachi FE S-4000 or LEO 1530. In order to visualize the internal structures of the casted follicles the samples of interest were frozen at -18°C and cut by a cooled razor blade [10].

Vessels were classified according to their diameters and the shapes of their endothelial cell nuclei [15]. Budding, sprouting, and splitting of capillaries from pre-existing blood vessels were considered proliferative (angiogenic) features [10-12]. The number of angiogenic structures in the inner vascular layer were counted according to Macchiarelli et al. [11].

#### Quantification of the vascular parameters

For the quantification analysis, images of the specimens were acquired by SEM at an accelerating voltage of 12 kV, a working distance of 15.2 mm and a magnification of 150×. Images were then imported into a morphometry system (Soft Imaging System GmbH-AnalySIS; Zeiss) in order to quantify vessel diameters and bifurcation angle (d0, d'1, d'2, d1 and d2 diameters of parent vessels, branches at the point of bifurcation or immediately after the bifurcation, respectively), in accordance with Djonov et al. [22]. These data were used to calculate the vascular branching degree: the bifurcation exponent Δ



the area ratio β



and the asymmetry coefficient γ



The vessel diameters were calculated in all the follicular plexuses. On the contrary, Δ, β, γ, and Δ', β and γ' (values immediately after, and at the point of bifurcation, respectively) were calculated only in the microvasculature > 40 μm since in the smaller vessels the blood flow does not respond to the Poiseuille's law [22-24]. For this reason, it was possible to perform the vascular geometric relations only in the middle plexus.

Finally, even if the casting method mimics the existing vessel morphology, it is known that for the smallest diameter vessels of 30 μm the polymerization process is subjected to a diameter distortion. Consequently, in the blood vessels with a diameter < 30 μm it was applied a correction factor of r = 1.369^(1/0.9606), were r is the radius of resin fixed vessels of the cast [25].

### Statistical analysis

Analyses were carried out on a total of 18 PreOFs, 22 EPerOFs and 20 LPerOFs.

Data obtained from different follicular stages, analyzed by LM, were assessed for Gaussian distribution. Successively, the values were compared using ANOVA test, and considered significant for P < 0.05. Analogously, the ANOVA test was used to compare the detected angiogenic structures.

The data obtained by SEM of VCC, since did not follow a Gaussian distribution, were represented as harmonic means (plus 25° and 75° percentile) and the comparison were carried out using the Mann-Whitney U-test after the Arctan transformation of data [22]. The differences were considered significant for P < 0.05.

## Results

### Group 1: preovulatory follicles (60 h after eCG)

#### Histological investigation

**Figure 1:**
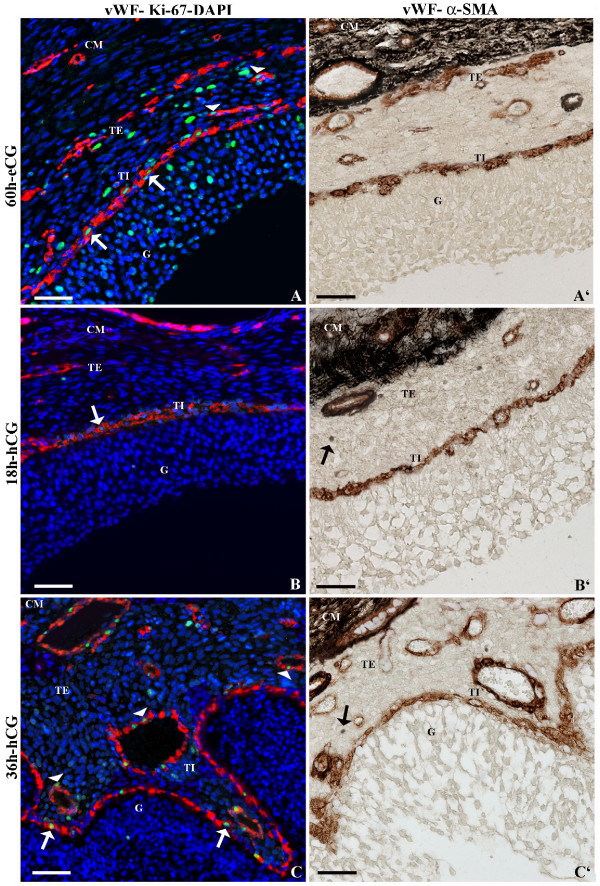
**Representative micrograph of immunostaining for vWF, Ki-67 and α-SMA in pre- and periovulatory follicles**. In the left panel (A, B, C) intrafollicular distribution of vWF (red stain), an endothelial marker, and Ki-67 (green stain), a cellular proliferative marker that were revealed using a double immunohistochemical technique. The cell nuclei were counterstained with DAPI (blue stain) to visualize the tissue morphology and to identify the follicular compartments. In the right panel (A', B', C') intrafollicular distribution of vWF (brown stain) and α-SMA (black stain) was assessed, by using a double immunohistochemistry. Preovulatory follicles were isolated 60 h after eCG (A, A'), and periovulatory follicles were isolated 18 h (B, B') and 36 h after hCG (C, C'). White arrows and white arrowheads indicate proliferating endothelial cells in the inner and outer network, respectively. In periovulatory follicles black arrows indicate single α-SMA immunopositive cells. G = granulosa compartment; TI and TE = inner and external (outer) theca compartment, respectively; CM = capsule of smooth muscular fibres. Bar = 100 μm.

The histological sections of porcine preovulatory follicle (PreOFs) revealed a compactness in the avascular granulosa layer. Generally, the granulosa compartment comprised fewer than 15 cell layers. The number of granulosa layers was consistent throughout the follicle, giving a "smooth" appearance to the granulosa-antral boundary. The inner theca layer was generally thin, with roundish cells running in parallel to the basal membrane, while the outer theca showed smaller spindle-shaped cells (Fig. 1A). At the periphery of the theca layer smooth muscle cells formed a conspicuous investment of concentric layers of stretched and elongated elements ("capsule of smooth muscle cells" [26] (Fig. 1B).

#### vWF

In preovulatory follicles, vWF staining evidenced two concentric blood vessel networks connected to each other by anastomotic vessels: i) an inner network made of relatively small diameter vessels, directly laying on the basal membrane, and ii) the outer one representing approximately the 57% of the total VA (Fig. 1A, 1A'; Table 1).

**Table 1:** Vascular Area (VA) recorded in the pig pre-(PreOF) and periovulatory (EPerOF-LPerOF) follicles.

	**PreOF (60 h-eCG)**	**EPerOF (18 h-hCG)**	**LPerOF (36 h-hCG)**
**Total VA (μm^2^)**	6578.21 ± 1125.21^a^	3925.42 ± 889.75 ^b^	8209.39 ± 2784.25 ^c^
**Inner network (μm^2^)**	2785.45 ± 199.87	2399.22 ± 196.32	2604.13 ± 247.89
**Outer network (plus anastomotical vessels) (μm^2^)**	3792.76 ± 801.22^a^	1526.20 ± 545.69 ^b^	5605.26 ± 1005.96 ^c^

The values are expressed as mean ± S.D.

^a^, ^b^, ^c^. Values with different superscripts within each line of parameters indicate data significantly different at least for *P *< 0.05.

Blood vessels were also evident inside the smooth muscle layer (Fig. 1A, 1A').

#### vWF-Ki-67

The endothelial cell marker vWF (red stain) and the proliferating cell marker Ki-67 (green stain), were specifically used to identify the proliferating endothelial cells, visible in the inner and outer vascular networks (Fig. 1A; Table 2).

**Table 2:** Endothelial Proliferation index (EPI) recorded in the pig pre-(PreOF) and periovulatory (EPerOF-LPerOF) follicles.

	**PreOF (60 h-eCG)**	**EPerOF (18 h-hCG)**	**LPerOF (36 h-hCG)**
**EPI (%)**	11.43 ± 3.11^a^	0.35 ± 0.24 ^b^	14.28 ± 2.98 ^a^

The values are expressed as % ± S.D.

^a^, ^b^. Values with different superscripts within each line of parameters indicate data significantly different at least for *P *< 0.05.

#### vWF-α-SMA

Endothelial cells (vWF brown positivity) of inner and outer network showed also α-SMA immunopositivity (black) (Fig. 1A'). Large vessels from the outer theca displayed α-SMA immunopositivity at their periphery (Fig. 1A').

A circular layer of smooth fibers was evident at the periphery of theca layer (Fig. 1A').

### Vascular corrosion cast

#### Light microscopy

The surface of the VCC of the ovary revealed numerous rounded or ovoid structures located within the ovarian cortex, representing the vascular plexuses of the different types of follicles (data not shown).

#### SEM

**Figure 2:**
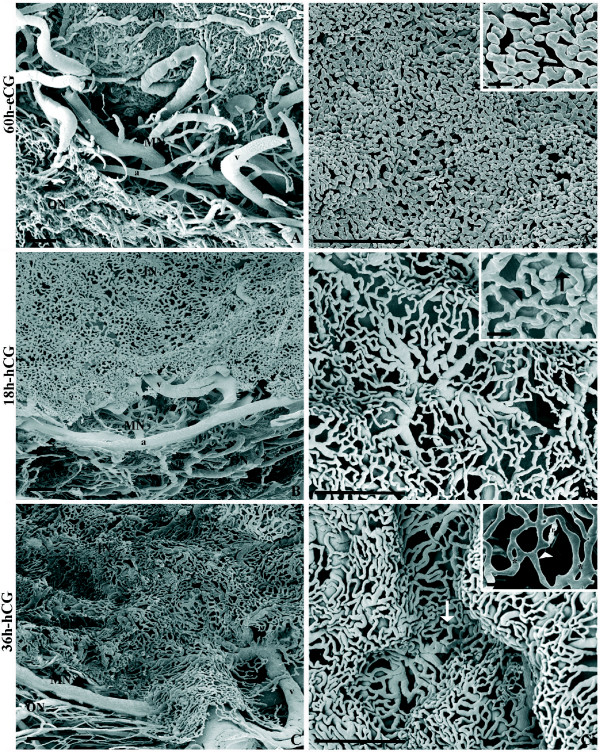
**Representative micrograph of SEM of VCC (fractured samples) in pre- and periovulatory follicles**. In the left panel (A, B, C) the vascular plexus (with inner, middle and outer networks) is showed. The right panel (A', B', C') shows the microvasculature of the inner capillary network. Inserts in A', B', C' illustrates the angiogenic figures (arrowhead = budding; arrows = sprouting; asterisk = infolding-intussusceptive). Preovulatory follicles were isolated 60 h after eCG (A, A'), and periovulatory follicles were isolated 18 h (B, B') and 36 h after hCG (C, C'). c = capillaries; a = arteriole; v = venule; IN, MN and ON = inner, middle, and outer network, respectively. r = resin leakage artefact. Bar = 100 μm. Bar in insert panel = 25 μm.

The architecture of the microvasculature of follicles with a diameter > 6000 μm appeared composed by a spherical basket-like configuration presenting a central empty area (corresponding to the region occupied by the granulosa layer, oocyte and antrum cavity before corrosion). The vascular plexus presented three layers: inner, middle and outer plexus (Fig. 2A).

The inner layer (about 60 μm in thickness) was constituted by a homogeneously dense capillary network, delimiting the inner wide cavity, and characterized by closely packed, small and short, capillaries (Fig. 2A'), with a small diameter (Table 3). The frequently observed angiogenic figures were budding and splitting from pre-existing capillaries (Fig. 2A' insert panel). Differences in the number of angiogenic structures between apical, equatorial and basal regions of the inner network were detected (Table 4).

**Table 3:** Vessel diameters recorded in the pig pre (PreOF)- and periovulatory (EPerOF-LPerOF) follicles.

	**PreOF (60 h-eCG)**	**EPerOF (18 h-hCG)**	**LPerOF (36 h-hCG)**
**Inner vascular plexus**	7.08 [6.84–7.98]	6.41 [4.69–7.34]	8.25 [6.34–12.67]
**Middle vascular plexus**	46.23 [43.87–72.20]	44.40 [39.60–61.39]	66.62 [63.37–73.27]
**Outer vascular plexus**	11.61 [7.31–16.45]	11.09 [7.92–23.76]	11.36 [9.50–19.01]

The values are expressed as harmonic mean with the 25° and 75° percentile since all values did not display a Gaussian distribution (P > 0.05).

**Table 4:** Number of angiogenic figures recorded in the pig pre-(PreOF) and periovulatory (EPerOF-LPerOF) follicles.

	**PreOF (60 h-eCG)**	**EPerOF (18 h-hCG)**	**LPerOF (36 h-hCG)**
**Apical region**	1.05 ± 0.49	1.97 ± 0.78	n.d.
**Equatorial region**	13.25 ± 1.45 ^a^	4.76 ± 0.99 ^b^	11.45 ± 1.57 ^a^
**Basal region**	3.01 ± 0.88	4.02 ± 1.01	3.91 ± 1.56

The values are expressed as mean ± S.D.

^a^, ^b^. Values with different superscripts within each line of parameters indicate data significantly different at least for *P *< 0.05.

n.d. = not determinable

The middle meshwork, of about 300 μm in thickness, was composed of arterioles and venules (Fig. 2A; Table 3). Vessels from the middle layers supported the inner plexus (Fig. 2A).

The outer plexus, of about 200 μm in thickness, was characterized by capillaries (Table 3) disposed at different levels (Fig. 2A).

#### Vascular geometric relations

Geometric relations of the area ratios (β, β'), the asymmetry ratios (γ, γ ') and the bifurcation exponents (Δ, Δ') of vessels were calculated at points (β', γ' and Δ') and immediately after (β, γ and Δ) the bifurcation of a blood vessel. These measures were calculated only in blood vessels with a diameter > 40 μm, thus only in the middle network (Table 5). The branching angles ranged around 45° (Table 5).

**Table 5:** Vascular geometric relations in the vessels of middle plexus of pig pre- and periovulatory follicles.

	**PreOF (60 h-eCG)**	**EPerOF (18 h-hCG)**	**LPerOF (36 h-hCG)**
**β'**	1,029 [1.029–1.640]	0,972 [0.910–1.088]	0,914 [0.869–1.259]
**γ'**	0,816 [0.733–1.000]	0,691 [0.600–0.833]	0,725 [0.658–1.000]
**Δ'**	2,142 [1.8151–2.288]	1,977 [1.811–2.024]	1,871 [1.666–2.199]
**β**	1,138 [1.017–1.569]	0,967 [0.813–1.199]	0,880 [0.742–1.438]
**γ**	0,773 [0.713–1.000]	0,644 [0.625–0.667]	0,758 [0.668–1.000]
**Δ**	2,113 [2.061–2.333]	1,962 [1.507–2.392]	1,891 [1.401–2.286]
**Branching angles (°)**	49.1 [41.0–65.5]	46.3 [33.0–52.1]	50.00 [38.0–72.3]

Vascular parameter degrees were valuated at points (β', γ', and Δ') and immediately after (β, γ, and Δ) vessels bifurcation.

The values are expressed as harmonic mean with the 25° and 75° percentile since all values did not display a Gaussian distribution (P > 0.05).

β, β' = area ratio; γ, γ' = asymmetry ratio; Δ, Δ' = bifurcation exponent.

### Group 2: early periovulatory follicles (18 h after hCG)

#### Histological investigation

The follicular morphology was similar to group 1 except for a beginning of functional luteinisation in granulosa and theca layers.

#### vWF

Compared to PreOFs, group 2 showed a reduced VA in the outer network (about 40%; P < 0.05), while the inner one resulted unaltered (Fig. 1B, 1B'; Table 1).

Blood vessels were observed in the capsule of smooth muscle layer (Fig. 1B, 1B').

#### vWF-Ki-67

At the beginning of the periovulatory phase, the simultaneous staining for vWF and Ki-67 evidenced a reduced EPI (Table 2, P < 0.05; Fig. 1B).

#### vWF-α-SMA

As showed in group 1, this double-immunostaining evidenced α-SMA immunopositivity (black) around the vWF-positive cells (brown) of the inner and outer networks. Moreover, in the outer network, large blood vessels showed an α-SMA-positive coverage at their periphery (Fig. 1B'). Single cells in the follicular wall displayed α-SMA immunopositivity (Fig. 1B').

Also in the EPerOFs a concentric layer of smooth fibres was recorded (Fig. 1B').

### Vascular corrosion cast

#### Light microscopy

The ovarian morphology in EPerOFs did not change compared to PreOFs.

#### SEM

In this phase, the inner network evidenced comparable thickness (about 60 μm) and blood vessel diameter to group 1 (P < 0.05; Table 3). By contrast, in the inner layer of EPerOFs, an evident elongation of the capillaries, accompanied by a decreased compactness was showed (Fig. 2B'). Budding and sprouting were the angiogenic figures visible (Fig. 2B', insert panel) also at this stage. Differences in their number were recorded among the three inner network regions. However, respect to group 1, in the inner equatorial region the angiogenic figures significantly decreased (P < 0.05; Table 4).

In the medium layer (thick about 300 μm) blood vessels resulted similar to periovulatory follicles (Table 3). Arterioles and venules from the middle plexus showed a low density (Fig. 2B). However, these blood vessels were visible underneath the gaps of the inner network (Fig. 2B, 2B').

The outer plexus, of about 200 μm in thickness, showed several blood vessels similar to group 1 (Table 3; Fig. 2B).

#### Vascular geometric relations

As shown in Table 5, the area ratios (β, β'), the asymmetry ratios (γ, γ') and the bifurcation exponents (Δ, Δ') of the middle plexus blood vessels were similar to group 1 (P > 0.05). The branching angles did not record any significantly difference respect to group 1 (P > 0.05; Table 5).

### Group 3: late periovulatory follicles (36 h after hCG)

#### Histological investigation

Close to ovulation, pig follicular sections showed evident changes in the follicle wall. The luteinized granulosa cells appeared to be "streaming" into the antrum, with fewer connections to the other cells or to the basal membrane. The basal membrane presented protuberances ("infolding"; [27]) into the antrum. The inner theca had variable characteristics associated with the degree of morphological changes visible in the granulosa layer. In fact, as evidenced by the presence of several projections from the inner theca following the granulosa compartment, the inner spherical morphological (i.e. "smooth") aspect was lost (Fig. 1C, 1C').

The capsule of smooth muscle cells did not change when compared to the other groups of follicles (Fig. 1C').

#### vWF

The total VA significantly increased thus overcoming the extension recorded in group 1 (Fig. 1C, 1C'; Table 1). Particularly, while the inner network remained unaltered (P > 0.05; Table 1), the outer layer reached ≅ 70% of the total VA (Table 1). Besides, anastomotical vessels increased their extension as well as their lumina (Fig. 1C, 1C').

Blood vessels were also recorded in the smooth muscle compartment (Fig. 1F).

#### vWF-Ki-67

Close to ovulation the simultaneous staining for vWF and Ki-67 evidenced an increased EPI *vs *group 2 (Table 2; Fig. 1C), while showed unaltered values *vs. *group 1 (Table 2).

#### vWF-α-SMA

Perivascular cells (demonstrated by α-SMA immunopositivity) were localized both in the theca tissue and in the cells associated with the blood vessels of the inner and outer networks (Fig. 1C'). In detail, in the inner theca vessels (near to the basal membrane) the black immunostaining was "chain-like" around the brown immunomarking, while in the outer theca large blood vessels with a well defined α-SMA-positive coverage were evident at their periphery (Fig. 1C').

A circular layer of smooth fibers was also evidenced in LPerOFs (Fig. 1C').

### Vascular corrosion cast

#### Light microscopy

In the largest follicles, blood vessels were not visible in the apical region, thus evidencing a "cup-like" shape (data not shown).

#### SEM

**Figure 3:**
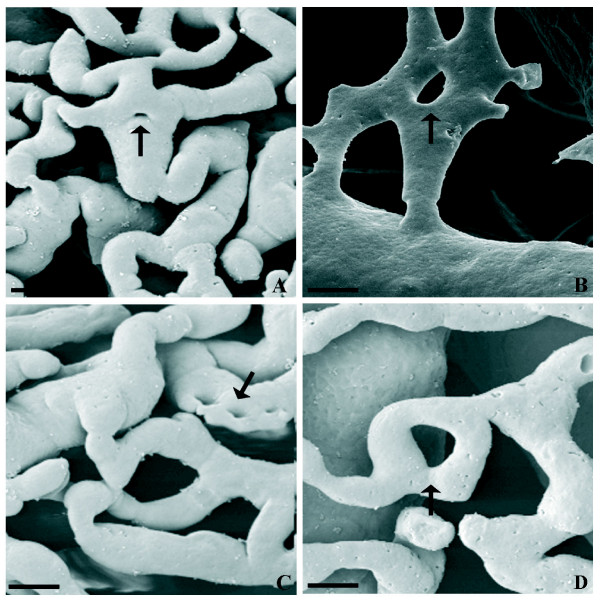
**Representative micrograph of different morphological pillar features in the LPerOF observed at SEM of VCC**. A) and B) show pillars in the centre of the vessel or shortly distant from the bifurcation, respectively (intussusceptive branching remodelling (IBR). C) show long parallel row of pillars in the longitudinal folds of the endothelium (intussusceptive arborisation; IAR). D) show pillar formation within the capillary bed (intussusceptive microvascular growth; IMG). Black arrows indicate pillars. Bar = 10 μm.

The inner microvasculature architecture of LPerOFs resulted similar to groups 1 and 2, both in thickness (about 60 μm) and in vascular diameters (P > 0.05; Table 3). However, differently to them, this vascular plexus showed an undulated aspect (infolding) with numerous and large gaps (Fig. 3C'). In detail, the middle network blood vessels lifted in folds the inner plexus (Fig. 3C). The inner layer displayed several angiogenic figures (similarly to group 1), differently distributed among the different regions (the uncasted apical region was not determinable; Table 4), and mainly constituted by sprouting (budding, splitting) and non-sprouting (intussusceptions) angiogenesis (Fig. 3D' insert panel). In particular, the inner vascular plexus showed the formation of numerous meshes (diameter < 3 μm) and transcapillary pillars raging from 3 to 20 μm. The intussusceptive pillar appeared shortly distant from the bifurcation or in the centre of the vessel in some circumstances, while in others holes appeared within the capillary bed, otherwise long parallel rows of pillars appeared in longitudinal folds of the endothelium (Fig. 3).

Due to the presence of several folds, the thickness of the middle layer ranged from 200 to 500 μm with the blood vessels showing a diameter similar to the other groups (Table 3).

The outer network thickness was of about 200 μm. In this plexus, vessel diameter (Table 3) and distribution were similar to groups 1 and 2 (Fig. 2C).

In the vascular plexus of the LPerOFs resin leakages were randomly present (Fig. 1C').

#### Vascular geometric relations

In this phase geometric relations of the vascular parameters were similar to the other groups (P > 0.05; Table 5) as well as branching angles values (P > 0.05; Table 5).

## Discussion

The present research for the first time compares IHC and SEM of VCC methods allowing to better investigate the physiological evolution of angiogenesis during the pig periovulatory phase. The results obtained evidence how, in terms of angiogenesis, the periovulatory follicle is a highly dynamic structure. In fact, the EPerOFs are in a quiescent status of angiogenesis, as evidenced, compared to PreOFs, by a reduced VA, EPI, and the number of angiogenic figures in the equatorial region. Moreover, a decreased inner layer compactness accompanied by a parallel elongation of the capillaries was detected. On the contrary, close to the ovulation (36 h after hCG), LPerOFs become morphologically transformed with an intense vascular remodeling and an increased vascular extension by EPI, sprouting and non-sprouting angiogenesis. These data allow to hypothesize the metamorphosing nature of the EPerOF that in only few hours will evolve into the highly vascularized structure (LPerOF), probably in order to sustain the corpus luteum development after ovulation.

Particularly, even if quiescent, in EPerOF the persistence of an active inner capillary network evidenced the importance of a correct trophic supply of oxygen and metabolites both to the avascular granulosa compartment and to the germinal cell [28], that in this phase is ending the first meiotic division and starting the second one.

As demonstrated in other species [18,29], the follicular wall of EPerOFs, presents perivascular cells. Their presence could be linked to the complex ontogeny that shows different and specific functions, all necessary for the angiogenic metamorphosis of the EPerOF. In fact, for example, in the brain pericytes can act as macrophages precursor cells [30], while in the liver they regulate the remodeling of the extracellular matrix [31]. However, it is also known that pericytes can be mainly related to the blood vessels stabilization and hemodynamic processes [32,33].

Approaching to ovulation, the LPerOF morphology resulted completely modified. For the first time, several theca folds appeared, probably precursors of the centripetal processes that will support the early stages of the corpus luteum development [34]. Secondarily, VA and EPI increased and the vascular architecture was completely reorganized since large blood vessels appeared near the basal membrane, with an α-SMA immunopositive coverage. The presence of smooth muscle coverage in the small venules/arterioles could be probably necessary to regulate the blood flow and their contractility contributing to the control of the ovarian function [1].

Although perivascular cells have been rarely identified in the capillaries [32], in this report they were also visible in the inner network of periovulatory follicles. Probably this location is determined on a functional basis, even as it is still unknown how pericytes choose their exact location on the vessel wall. In fact, it has been noted that they preferentially cover endothelial cells junctions specifically during inflammation [32], a process that similarly occurs during ovulation [1]. It is noteworthy how, the angiogenic stimuli brought by perivascular cells [18,32,33,35] could be important in those follicles undergoing transformation into the most vascularized structure of the organism, the corpus luteum [1].

The SEM analysis of LPerOFs evidenced sprouting and non-sprouting angiogenesis. Interestingly, there was no morphological evidence of the transcapillary pillar formation before the late periovulatory phase, suggesting that intussusception did not occur before this developmental stage.

In particular, in contrast to sprouting, intussusceptive angiogenesis a) occurs in the virtual absence of endothelial cell proliferation, b) is achieved at low vascular permeability levels, and c) requires only 4–5 h for completion [36]. All of them are fundamental conditions, considering how quickly the follicle will ovulate to become a corpus luteum [36,37]. In addition, the different morphological features of pillars recorded in LPerOFs suggest different outcomes. In fact, the continuous pillar formation and growth within the capillary bed leads to intussusceptive microvascular growth (IMG); rows of round pillars that changed shape to acquire a slit-like configuration and then merged each other to form small vessels leads to intussusceptive arborisation (IAR); pillar formation occurring within small vessels can guide to a vascular branches remodeling leads to intussusceptive branching remodeling (IBR) [38]. IMG represents a general and ubiquitous mechanism of capillary growth where the vascular bed can undergo a rapid expansion without compromising vascular physiology or function [22,36,38-40]. IAR allows the formation of a feeding vascular tree [37,41], while, in IBR, the branching geometry of supplying vessels is adapted to optimize the changes in the hemodynamic condition of the vascular tree [16,38,39]. Numerous growth factors and cytokines, such as VEGFs, angiopioetins, FGFs, PDGFs, IGFs, TGFα, and β, as well as proteinases, and heparin, have been shown to posses angiogenic regulatory potential [2,6,42]. The majority of the studies have been conducted on sprouting angiogenesis. However, these factors are probably also involved in the control of non-sprouting angiogenesis [36]. Furthermore, the presence of these different angiogenic factors as well as the increased permeability of the follicular blood vessels close to ovulation [1], may explain the presence of the resin leakage detected in LPerOFs.

During the periovulatory period, no significant increase in blood vessel diameter was evidenced, thus supposing that the increased demand in terms of intrafollicular blood flow could be mainly related to angiogenesis. On the contrary, during the preovulatory period an extension of blood vessels was coupled both to angiogenesis and to vasodilatation of the existing capillaries [43].

However, the results of the present study on periovulatory follicles evidenced a great variability in the vascular diameter and a region-dependant distribution of the angiogenic figures. Similar findings were described in rabbits [44,45] by SEM of VCC, and in women by transvaginal color Doppler ultrasonography [46]. In particular, Brannstrom et al. [46] revealed marked regional differences in human follicular blood flow with a sustained increase in the basal and lateral follicular walls and a concomitant decrease in the flow of their apical region. These data were confirmed in this research because 36 h after hCG administration, the upper part of the follicles could not be observed: probably the diameter of thinnest blood vessels did not allow the resin to penetrate. These vascular changes are probably required for the follicle rupture: minor blood supply at the stigma is necessary for the subsequent ovulation [47].

In this work, the histological investigation of the pre- and periovulatory follicles showed a double concentric network, connected by anastomotic vessels, in the theca compartment. In the capsule of smooth muscle cells, blood vessels were also observed. On the contrary, SEM analysis of VCC evidenced that the vascular plexus was characterized by three layers, but it was impossible to define to which of the two concentric networks these structures belonged, because tissues were corroded. The comparison of the two methods – IHC and SEM of VCC – and the thickness of the layers, could allow us to assume that the inner and middle vascular plexuses observed by SEM correspond to the two concentric networks detected at the LM, while the outer vascular plexus evidenced by SEM represented the vessel layers observed in the smooth muscle compartment by histological investigation.

The analysis of the vascular geometric relations showed how the found values of bifurcation exponents Δ, Δ' (≅ 2) and area ratios β, β' (≅ 1) were in evident contrast with the Murray's law prediction. In fact, the Murray's minimum volume optimization theory defined Δ = 3. On the contrary, West-Brown-Enquist [WEB, [48]], representing the branching geometry as a fractal structure, calculated that when Δ = 2 and β = 1 the optimization criterion of minimum surface areas was satisfied (WBE model). Moreover, the Δ = 2 is in accordance with the interpretation of Woldember and Horsfield [49] that predicted the condition in which both drag and power loss are minimized. It is noteworthy that when Δ = 2 the mean blood velocity became independent from radius [50] since the velocity in a tube of cross sectional area A is:



v = mean velocity, V = blood volume, η = dynamical viscosity, R = radius, p'= pressure gradient (= dp/l), c = constant.

Again, in these conditions the area of parent vessel is identical to the sum of the areas of daughter vessels thus β ≅ 1 allows to postulate:



in agreement of WBE model.

The γ, γ' values of ≅ 0.75 indicated a moderate degree of asymmetry in the vascular structures demonstrating a moderate degree of vascular structure instability.

The branching angles mean values (45°–50°) in different follicular stages were compared with the theoretical prediction of Zamir's equations for asymmetrical (γ < 1) branching [51] that indicated the optimal angle values in respect of different optimization criteria in function of the asymmetry ratio. On this basis, the branching angles recorded in this work were only occasionally correlated with Zamir's predictions, indicating that the empirically founded branching angle values are not always in agreement with the optimal theoretical conditions.

The evaluation of these geometrical parameters consents to postulate that the development of periovulatory follicular vascular network is driven by the balance between the principles of optimality and the biological needs. In fact, in one hand the vascular design seems to respect the fractal hypothesis and the minimization of surface area criterion, while in the other one the limitations due to geometrical or biological reasons are evident (i.e. branching angle). Probably, the deterministic and random processes cooperate to design the vascular networks architecture, as stated by Kurz [50] for embryonic brain and spinal cord. This underlines that a mathematical approach has always to be integrated with the morphological and physiological findings, to improve the knowledge of a complex biological event such as the vascular remodeling in periovulatory follicles.

## Conclusion

The present work for the first time combines IHC and SEM of VCC techniques to study the vascular architecture remodeling in pig ovarian follicles during the periovulatory phase (from LH surge to ovulation) where the follicle, a limited blood supplied structure, get transformed in a well vascularized organ, the corpus luteum. This combination results in a powerful method allowing to investigate the extent, the morphological and morphometrical appearance of the follicular vasculature in a stage of great changes and remarkable reproductive importance.

The obtained results allow to hypothesize that, in terms of blood vessels remodeling, the pig periovulatory phase can be divided in two moments: an early and a late stage. In the early stage (18 h after hCG), a turn-off in follicular angiogenesis is observed. This quiescence represents the starting point for the subsequent metamorphosing process, necessary to transform the periovulatory follicle into the next physiologic structure. In fact, in the late stage (36 h after hCG), close to ovulation, the metamorphosed follicles rapidly and strongly turn-on its angiogenic activity to sustain a successful corpus luteum formation.

Our hypothesis can be sustained by the following observations: 1) in EPerOFs there is an essential vascular asset, represented by the maintenance of the inner vascular layer, probably in order to sustain the avascular granulosa compartment and the maturing oocyte, with few proliferating endothelial cells and rare angiogenic figures; 2) the presence of perivascular cells could sustain the vascular remodeling necessary to the corpus luteum formation; 3) in LPerOFs high level of angiogenesis are accompanied to tissues and vascular reorganization: the follicular morphology looses the typical roundish aspect acquiring an undulated one. This seems mainly consequence of the inner vascular plexus infolding toward the antrum, made by arterioles- or venules-like from the middle network near to basal membrane. The turning-on of angiogenesis in LPerOFs is also demonstrated by a newly endothelial cell proliferation (similarly to PreOFs) and an increased endothelial area by sprouting and non-sprouting angiogenesis. Particularly, it is interesting to underline that the non-sprouting angiogenesis is present only in this stage and it is characteristic of structures in rapid neovascularization.

The analysis of the vascular geometric relations allowed to hypothesize that the periovulatory vascular remodeling happens with the "maximum output with minimal outflow of energy".

The knowledge of follicular angiogenesis is of fundamental importance not only to understand the mechanisms that ensure the reproductive success, but also in order to better clarify the angiogenic process in adult tissues. In this context, folliculogenesis becomes a very important experimental model for the cyclic nature of angiogenesis and because of its reproducibility by validated hormonal pharmacological treatments [5,6,17]. Finally, it is important to point out how the use of the ovarian follicles allows to overcome many typical problems correlated with the use of the transgenic models [52], often related to the creation of biological artifacts that inevitably require a physiological confirmation.

## Competing interests

All the authors give assurance that the work is not under consideration by any other journal, and they declare there are no competing interest; financial or otherwise, in relation of this work.

## Authors' contributions

AM and MGP designed, directed the study and wrote the manuscript. AM, MGP, VR, NB, CR, ODG have made contribution to acquisition of data. In particular AM, MGP, VR carried out the IHC and SEM of VCC studies. CR and ODG participated in the IHC and VCC studies. NB performed the vascular geometric relations and statistical analysis. PB has been involved in drafting the manuscript. SAN participated to the interpretation of SEM data. GM and BB participated in the interpretation of data and critically revised the manuscript. All authors read and approved the final manuscript.
